# Supplementary material for: Transcriptomic Insights into Mechanisms of Early Seed Maturation in the Garden Pea (Pisum sativum L.)
Source: Cells. 2020 Mar 23;9(3):779. doi: 10.3390/cells9030779 (PMC7140803; doi:10.3390/cells9030779)
Supplement: Supplementary file 1 [file cells-09-00779-s001.zip › Supplementary materials/FileS2.zip!/FileS1/Summary.html]

Summary of Full transcriptomic landscape dataset


# General Information

Number of samples
:   24

Number of groups
:   6

Number of genes
:   27836

ID type of genes
:   not defined

Dimension of the SOM
:   40 x 40

Date
:   Чт дек 12 16:48:01 2019 MSK

Analyst
:   reverend\_casy

oposSOM version
:   2.4.0

# Results

## General

- Data Distribution (PDF)
- Chromosome-wise gene expression heatmap (PDF)

## SOM Analysis

These reports comprise the SOM portraits in standard and alternative
color scales, as well as supporting maps and profiles which provide
supplementary information about the 1st level SOM.

- 1st Level SOM Expression Portraits (PDF)
- Alternative Color Scales: absolute expression, WAD, loglogFC (PDF)

- Supporting Maps (PDF)

- Entropy Profiles (PDF)
- Topology Profiles (PDF)

- Localization of the genes within the SOM (CSV)

## Sample Summaries

Summary page for the individual samples.

- Sample Reports (HTML)

## Sample Similarity Analyses

Sample similarity analyses based on expression module data and metagene data using different metrics and algorithms.  
  
Euclidean Distance based approaches:

- Sample SOM (PDF)
- t-SNE dimension reduction (PDF)
- Phylogenetic clustering using neighbor joining algorithm (PDF)
- Expression heatmaps and hierarchical clustering dendrograms (PDF)

Correlation based approaches:

- Correlation spanning tree connecting all samples (PDF)
- Correlation backbone, a 2-nearest-neighbor graph (PDF)
- Correlation network connecting samples with r > 0.5 (PDF)
- Pairwise correlation maps (supervised & clustered orderings) (PDF)

Component Analysis:

- Independent Component Analysis: 2d-ICA, 3d-ICA (PDF)

## Expression Module Analysis

Different criteria of spot module definition such as overexpression or
mutual correlations between the metagenes where applied.
The reports comprise integrated portraits, functional analyses.

- Spot Reports (HTML)

Analyses based on PAT-wise aggregated data, including clustering dendrogram,
portraits and assotiation to the sample groups.  
PATs were defined using *dmap* approach.

- PAT Report (PDF)

## Group Analyses

Analyses based on group-wise aggregated data, including portraits,
clustering and functional analyses.

- Group Analysis Reports (HTML)

## Pairwise Differences Analyses

Analyses based on pairwise comparisons, including portraits,
clustering and functional analyses.

- Differences Analysis Reports (HTML)
